# Supplementary material for: Splice-Junction-Based Mapping of Alternative Isoforms in the Human Proteome
Source: Cell Rep. Author manuscript; Available in PMC 2020 Jan 15. (PMC6961840; doi:10.1016/j.celrep.2019.11.026)
Supplement: 2 [file NIHMS1546469-supplement-2.zip › DF1/PXD006675/MitralValve/MitralValve_3_TNXB_MGPLSVVIVTVAR.pdf]

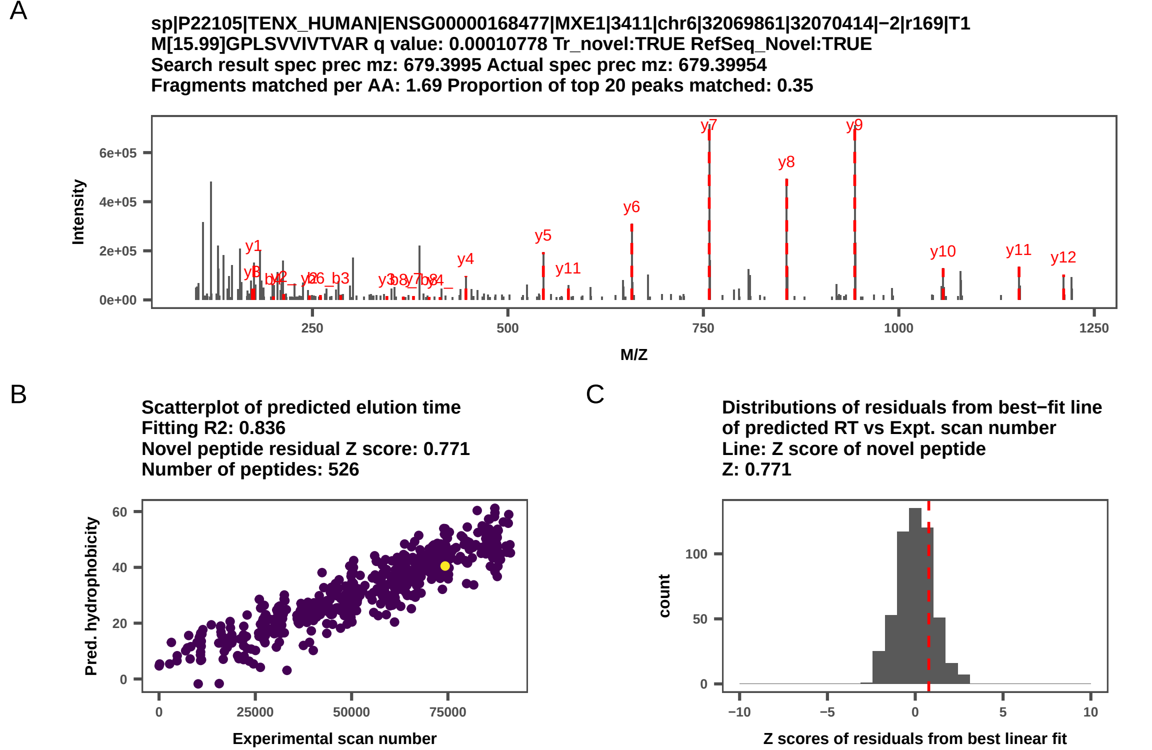
A
sp|P22105|TENX_HUMAN|ENSG00000168477|MXE1|3411|chr6|32069861|32070414|−2|r169|T1
M[15.99]GPLSVVIVTVAR q value: 0.00010778 Tr_novel:TRUE RefSeq_Novel:TRUE
Search result spec prec mz: 679.3995 Actual spec prec mz: 679.39954
Fragments matched per AA: 1.69 Proportion of top 20 peaks matched: 0.35
Intensity
M/Z
y7
y9
y8
y6
y5
y1
y4
y11
y10
y11
y12
B
Scatterplot of predicted elution time
Fitting R2: 0.836
Novel peptide residual Z score: 0.771
Number of peptides: 526
Pred. hydrophobicity
Experimental scan number
C
Distributions of residuals from best−fit line
of predicted RT vs Expt. scan number
Line: Z score of novel peptide
Z: 0.771
count
Z scores of residuals from best linear fit
